# Disseminated Cutaneous, Osteoarticular, and Tubulointerstitial Sporotrichosis in an Immunosenescent and Diabetic Host: A Case Report

**DOI:** 10.1155/crdm/7955710

**Published:** 2025-11-26

**Authors:** Juan Esteban Velez-Hernandez, Natalia Giraldo, Erika Andrea Sánchez-Cifuentes, María del Pilar Jiménez-Alzate, Fernando Lopez-Urbano

**Affiliations:** ^1^Department of Internal Medicine, University of Antioquia, Medellín, Colombia; ^2^Hospital Alma Mater, Medellín, Antioquía, Colombia; ^3^Clínica CES, Medellín, Antioquía, Colombia; ^4^Dermatology Department, University of Antioquia, Medellín, Colombia; ^5^Medical Mycology Group, University of Antioquia, Medellín, Colombia

**Keywords:** diabetes mellitus, immunosenescence, interstitial nephritis, septic arthritis, sporotrichosis

## Abstract

**Introduction:**

Disseminated sporotrichosis is an uncommon presentation of this infectious disease, primarily observed in immunocompromised patients. Here, we present a case of disseminated sporotrichosis occurring in an immunosenescent patient.

**Case presentation:**

An 81-year-old man presented to our clinic with skin ulcers in the scalp, back, right arm, and both legs, knee arthritis, and acute kidney injury. After skin biopsy and synovial fluid analysis, the diagnosis of disseminated sporotrichosis was made using fungal culture and in-house PCR. The only factor that favored immunocompromise was immunosenescence.

**Conclusion:**

This case of disseminated sporotrichosis highlights its rare occurrence in a patient with controlled diabetes and immunosenescence. Advanced diagnostics confirmed *Sporothrix schenckii* as the causative agent, and itraconazole treatment led to significant improvement.

## 1. Introduction

Sporotrichosis is a fungal infection caused by species of the thermally dimorphic fungus *Sporothrix* spp., which includes members of the pathogenic clade and, to a lesser extent, some species from the environmental clade of the genus *Sporothrix*. Although *Sporothrix* spp. is considered a cosmopolitan fungus, and most cases of sporotrichosis occur in tropical and subtropical regions of Latin America, Africa, and Asia [1].

Sporotrichosis is a subacute or chronic implantation mycosis, typically following the traumatic inoculation of infectious propagules—conidia from the mycelial phase—into the skin, mucosa, or osteoarticular sites. These propagules are released into the environment and, upon entering the human body, undergo a phase transition into the yeast form at 35°C–37°C. Less frequently, infection can occur via inhalation, leading to pulmonary disease in both immunocompetent and immunocompromised individuals [2].

The disease commonly affects individuals engaged in agricultural or outdoor activities (e.g., floriculture, horticulture, gardening, mining, or hunting) that involve contact with decaying plant material contaminated with the fungus. This has earned sporotrichosis the colloquial names “rose gardener's disease” or “rose handler's disease.” In endemic regions, sapronotic (plant-associated) transmission mainly involves *Sporothrix schenckii* sensu stricto and *Sporothrix globosa*. However, zoonotic transmission via *Sporothrix brasiliensis*—particularly through deep scratches and bites from infected cats—has been associated with more severe clinical presentations in humans. This mode of transmission has caused the largest recorded outbreak of sporotrichosis, which continues to spread across multiple regions of Brazil and into neighboring countries [3].

Feline sporotrichosis is unique among endemic dimorphic fungal infections due to its direct transmission in the yeast phase. Infected cats often present with lesions harboring a high yeast burden, and transmission to humans may occur through scratches, bites, respiratory droplets (e.g., sneezing or coughing), or direct contact with secretions that breach integumentary barriers [4].

As with other endemic mycoses, sporotrichosis in immunocompromised individuals tends to present with more severe clinical manifestations. This increased severity is associated with diminished immune and inflammatory responses, higher fungal burdens, widespread dissemination, and elevated mortality rates. In cases of opportunistic sporotrichosis (OS), standard serological tests may produce false-negative results, and prolonged systemic antifungal therapy is often necessary [2].

Sporotrichosis primarily presents with cutaneous symptoms and only rarely progresses to disseminated disease. It is considered a spectral disease and is categorized into two main types: cutaneous sporotrichosis and extracutaneous sporotrichosis [5]. These categories include four distinct clinical forms: lymphocutaneous (LC), fixed cutaneous (FC), disseminated cutaneous, and extracutaneous. LC and FC forms are the classical and most frequently observed manifestations. Disseminated cutaneous and extracutaneous forms—considered more severe—typically occur in individuals with impaired cellular immunity [2].

Data from large outbreaks in regions such as China [6], Japan [7], Peru [8], and Brazil [9] indicate that severe forms occur in approximately 1.3%–9% of cases. The disseminated cutaneous form is a rare variant characterized by multiple noncontiguous skin lesions without internal organ involvement. It is often challenging to determine whether such cases result from dissemination from a single lesion or multiple independent inoculations. Extracutaneous or disseminated forms involve internal organs and systems, with affected sites including the skin, eyes, lungs, liver, kidneys, heart, central nervous system (CNS), and genitalia. Osteoarticular involvement may occur either via direct extension from a primary lesion or through hematogenous dissemination from the lungs [2].

This mycosis can affect anyone regardless of age, gender, or comorbidities, mostly depending on exposure [10]. Human immunodeficiency virus (HIV)/AIDS changes the natural history of sporotrichosis, and its opportunistic character depends on the immune status of the host. Comorbidities such as diabetes mellitus (DM), chronic alcoholism, steroid treatment, hematologic cancer, and organ transplantation have been sporadically described as risk factors for severe forms of the disease, and case reports have focused on unusual manifestations in these scenarios [2].

The most common underlying condition associated with sporotrichosis outbreaks is DM, followed by chronic alcohol use. Clinical presentation may vary between diabetic and alcoholic patients: Some exhibit disseminated cutaneous lesions, while others develop particularly severe, localized, and destructive lesions with a granulomatous appearance, suggesting enhanced pathogenicity in these localized cases [11–14].

The diagnosis of sporotrichosis is challenging due to the overlap of its clinical manifestations with those of other diseases. There are different tests that can be useful such as histopathological staining, direct and microbiological culture, or more specialized tests such as PCR [15, 16]. Staining with Grocott Methenamine Silver (GMS: 40% sensitivity), periodic acid-Schiff (PAS: 31% sensitivity), and Calcofluor White (CFW: 74% sensitivity) allows to observe in the tissue, globose-shaped blastoconidia, cigar-shaped cells (in 33% of cases), or round and oval within the cytoplasm of giant cells or in the center of asteroid bodies [17]. Direct examination with 10% potassium hydroxide of the lesion samples is not useful [18]. The gold standard is the microbiological culture such as Mycosel and Sabouraud agar [19]; however, fungal cultures may take 2 to 3 weeks to yield growth, and morphological typing allows identification only up to the genus *Sporothrix* and does not allow for discrimination between the different species [19, 20]. Molecular methods may improve the rapidity and accuracy of diagnosis; the use of group genes is recommended for distinguishing species including the CAL, BT2, and chitinase synthase gene (the CAL and chitinase gene amplification were used for this case); in addition, confirmation of species can be achieved by this method [21].

In this paper, we present a case of disseminated sporotrichosis occurring in an immunosenescent patient.

## 2. Case Presentation

An eighty-one-year-old man presented with a past medical history of hypertension and well-controlled Type 2 DM. He takes care of cattle, drinks unpasteurized milk, and spends much of his time in the woods.

**Figure 1:**
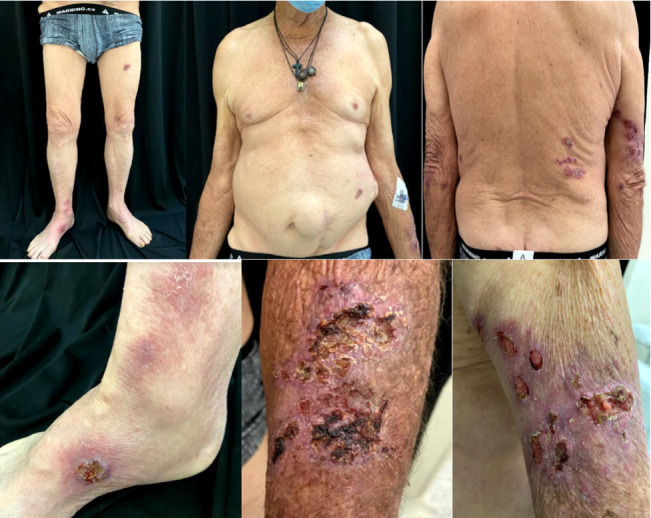
Multiple tender and ulcerated plaques, with erythematous edges, purulent secretion, and fibrin, measure between 2 and 3 cm and are located on the trunk, arms, legs, and scalp.

He reported a history of 4 months of skin ulcers, first spotted in the posterior region of the right arm associated with a previous injury with a tree stick, then more lesions appeared in the right arm, scalp, both feet, legs, and abdomen (Figure 1), and also he reported pain, erythema, and edema in the right knee.

Physical examination revealed adenopathy in the right axilla (1 × 1 cm), supraclavicular region (0.5 × 0.5 cm), and bilateral iliac chains (0.5 × 0.5 cm). The right knee and both ankles were swollen, red, and warm.

Skin lesions were sampled via punch biopsy (right upper back, superior right arm, left forearm, and right foot). The specimens underwent histopathological analysis (hematoxylin-eosin, silver stain, mucicarmine red, PAS, and Ziehl–Neelsen) and microbiological testing (cultures for anaerobic, aerobic, fungal, and mycobacterial species; Gram stain; India ink preparation; nested chitinase PCR for Sporothrix genus; and GeneXpert for Mycobacterium tuberculosis).

The intra-articular fluid of the right knee was obtained, and cytochemical, cultures, and polarized light tests were performed, all of which were negative. The Bengal rose test for *Brucella* spp was negative.

**Figure 2:**
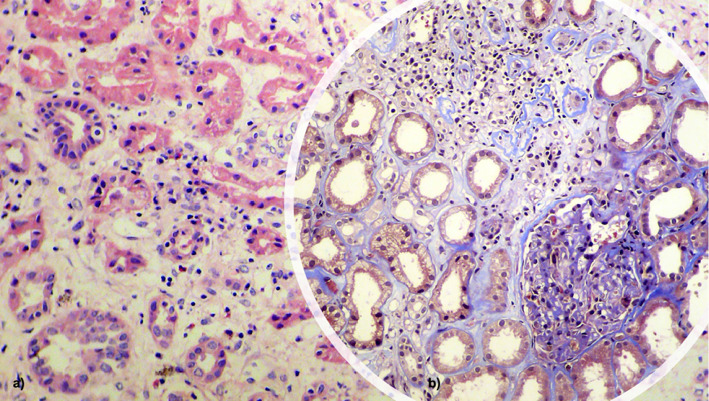
(a) 1 × 2 H.E. and (b) 1 × 200 Mason's trichrome. Description: Moderate interstitial mononuclear inflammatory infiltrate and edema. Some tubules with epithelial detachment and dilation of their lumen. Interstitial fibrosis and moderate tubular atrophy in 30% of the sample. Kappa and lambda light chains with linear trapping in glomerular and tubular bases and strong positivity in atrophic tubular casts. No findings of diabetic nephropathy. Conclusion: Chronic tubulointerstitial nephritis. Provided by Luis Fernando Arias, nephropathologist, University of Antioquia.

In the laboratory panel, creatinine was 5.2 mg/dL, BUN was 98 mg/dL, PTH was 58 pg/mL, phosphorus was 5.2 mg/dL, and ferritin was 1199 ng/mL, and renal ultrasound showed changes of chronic nephropathy; he did not require dialysis; nephrology was consulted and a renal biopsy was performed that showed patterns compatible with chronic interstitial nephritis (Figure 2) and lambda and kappa infiltrates in the parenchyma. Immunoglobulins, serum electrophoresis, urine electrophoresis, and immunofixation were normal.

**Figure 3:**
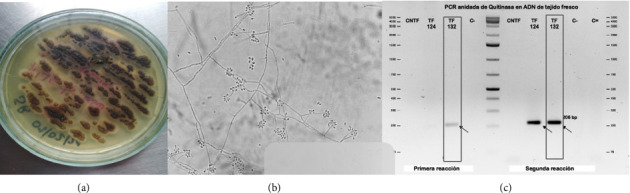
(a) On the plate, we see some membranous colonies of melanin pigment of sectorized distribution growing on Sabouraud. (b) Direct culture examination with lactophenol blue showing hyaline septate hyphae with conidiophores that produce conidia with an asynchronous botryous organization that gives the image of the typical daisy flower. (c) Chitinase-nested PCR which amplifies a 206 base pair sequence for the identification of fungi belonging to the *Sporothrix schenckii* complex (skin). The species *S. schenckii* sensu strict was confirmed by specific PCR from the DNA of the culture.

In cultures from the skin ulcers from different locations, *Sporothrix* spp grew. Also, chitinase-nested PCR was positive for *Sporothrix schenckii* complex (skin) (Figure 3). Thereafter, the same microorganism was obtained from the intra-articular fluid by fungal cultures. DNA extraction was done from fungal cultures, the calmodulin-nested *Sporothrix* species–specific PCR was done, and *Sporothrix schenckii* was confirmed as the causative species of the disease.

Treatment was guided by an infectious disease specialist with itraconazole 400 mg daily, and osteoarticular function, skin aspect, and symptoms improved after discharge [22].

## 3. Discussion

Sporotrichosis is the most common subcutaneous mycosis across the globe, and it is caused by dimorphic fungi belonging to the genus *Sporothrix* [23, 24]. This genus is comprised around 68 species, including *Sporothrix brasiliensis*, *Sporothrix globosa*, *Sporothrix mexicana*, *Sporothrix luriei*, *Sporothrix pallida*, and *S. schenckii sensu stricto* [25, 26]. *S. schenckii*, *S. brasiliensis*, and *S. globosa* are the most prevalent species [26] and are associated with human infections (pathogenic clade). The environmental clade, including *S. mexicana* and *S. pallida*, is rarely involved in human sporotrichosis but is responsible for opportunistic infections.

Because sporotrichosis is a dimorphic fungus, it takes two distinct forms: filamentous (in the saprophytic phase), when found in the environment or cultivated at 25°C, and yeast-like (in the parasitic phase), when cultivated at 37°C or inside the host. Environmental species typically exhibit low virulence because the conversion from mycelium to yeast, which successfully occurs among clinical clade species, is deficient in environmental species and results in few yeast-like cells [15].

After inoculation with the fungus, innate immune mechanisms are activated to directly control the infection and stimulate a specific immune response involving the Th1 and Th17 subsets of T lymphocytes [27, 28].

For sporotrichosis to develop in the host body, the conidia must undergo a dimorphic transformation when they come into contact with macrophages and become yeasts. The survival of the fungus is made possible by the low induction of a proinflammatory response and cell death induced by reactive oxygen species (ROS) [29]. This characteristic is crucial for the virulence of the fungus because the transition from hyphae to yeast results in changes to the cell wall, which exposes the antigenic components [30].

In addition, the ergosterol present in the fungus's cell membrane reacts with the hydrogen peroxide produced by macrophages to form ergosterol peroxide, which is one of the evasion mechanisms of the fungus [31]. Previous studies have shown that melanin, which is produced from phenolic compounds, such as l-3,4-dihydroxyphenylalanine (L-DOPA) [25], confers fungal resistance against phagocytosis and protects against UV radiation, temperature increases, and ROS [32, 33].

Macrophages play crucial roles in initiating, maintaining, and resolving the inflammatory response in the host. These cells act as regulatory effectors of the immune system by recognizing, phagocytosing, and processing the etiological agent for subsequent antigen presentation. After activation, macrophages phagocytose the invading pathogens and promote the production of proinflammatory cytokines, such as IL-6, IL-4, TNF-*α*, and IL-1*β*. These cytokines stimulate the phagocytic responses and promote a release of toxic agents that mediate the immune and inflammatory responses, such as nitric oxide (NO), which is highly cytotoxic to *S. schenckii* and is released at the beginning and end of infection [34–36].

Studies have shown the importance of Toll-like 2 (TLR2) and Toll-like 4 (TLR4) receptors in the innate immune response against *Sporothrix brasiliensis*. These receptors are responsible for detecting various components present in bacteria and fungi. The absence of TLR2 and TLR4 results in impaired phagocytosis and reduced levels of TNF-*α*, IL-6, IL-10, and nitric oxide, which could promote a persistent infection [37–39].

To prevent the entry of pathogens, the immune system uses various physical and chemical barriers, such as natural barriers, which include the skin and mucosa, and the production of cytokines, chemokines, and ROS. However, in individuals with DM, dysfunctions associated with inflammation result in the inability of the immune system to defend against invasive microorganisms and in increased susceptibility to infections [40, 41].

The clinical manifestations of the disease vary according to the host immune response, the yeast load, and the depth of inoculum, which ranges from localized skin lesions to cutaneous dissemination or systemic disease; in particular, systemic disease has been reported in immunosuppressed patients in whom the bones, joints, lungs, and CNS have been affected. Disseminated skin infections are characterized by generalized papules, nodules, plaques, and ulcers that may or may not follow lymphangitic pathways: These have been described mainly in patients with some type of immunosuppression (HIV, diabetes, alcoholism, and transplants, among others) [15].

Neely et al. [42] revealed that individuals with diabetes had a greater incidence of infections, including bacterial infections, such as osteomyelitis, pyelonephritis, cystitis, pneumonia, cellulitis, sepsis, and peritonitis, as well as fungal infections, compared to individuals without diabetes. Diabetes itself does not increase the risk of sporotrichosis. However, immunosuppression in individuals with DM can lead to the disseminated form of the disease due to immunosuppression in individuals due to failure to eliminate the pathogen [43] as well as poor infection treatment outcomes. Hyperglycemia in diabetes is thought to cause dysfunction of the immune system. Mechanisms include impaired cytokine production (IL-1B, IL-1, IL-2, IL-6, IL-10, IL-22, INF_*γ*_, CXCL1, CXCL2, and TNF-*α*), disruption in phagocytosis, leukocyte recruitment inhibition, reduced expression of TLRs, decreased opsonization, and immune cell dysfunction [40]. These combined impairments in our patients with diabetes may play a role in the disseminated infection, being the only risk factor besides immunosenescence that the patient had.

In general, older adults are more likely to contract more severe infections than younger adults due to their accumulation of comorbidities and immune senescence. The latter is characterized by a decline in immune response (both innate and acquired) and a proinflammatory condition known as “Inflammaging” involving an increase in interleukin-6 and tumor necrosis factor. Elderly frailty syndrome, which is more pronounced after 80 years of age, is a consequence of the decline of many physiological systems, resulting in vulnerability to changes in health status triggered by stressful events [44].

In a study of older patients with sporotrichosis published by Gomes et al. [44], they showed that the osteoarticular system was the most affected extracutaneous site (2.1%) due to the contiguity of the cutaneous lesions in the majority of cases. Among older people, the skin barrier is weakened due to the reduction in its layers and components [45, 46]. In this context, the progression of fungal infection may occur beyond the skin, and this is also supported by a larger and more virulent inoculum, which is typical of zoonotic transmission [47].

Our patient presented with tubulointerstitial nephritis; this is a renal lesion that typically causes a decline in kidney function and is characterized by an inflammatory infiltrate in the kidney interstitium that has been reported as a complication of different chronic infections (4%–10% of cases) including viruses (hantavirus and HIV), bacteria, parasites, and fungi. The kidneys, and particularly the glomeruli, are frequently affected by either the primary infectious process or the secondary immunological disease. Infections can lead to the formation of multiple autoantibodies, for example, rheumatoid factor, antinuclear antibodies (ANAs), antiphospholipid antibodies, and antineutrophil cytoplasmic antibodies (ANCAs) including ANCA-directed antigens other than myeloperoxidase (MPO) or proteinase-3 (PR-3). Glomerulonephritis has been associated with the formation of ANCA during the course of an infection, and it is associated with morbidity and mortality, regardless of its specific histological picture [48].

There is a report of a patient with chronic cavitary pulmonary sporotrichosis with elevated ANCA titers. Treatment of the pulmonary sporotrichosis with itraconazole resulted in the patient's clinical improvement and normalization of his ANCA levels [49].

To our knowledge, this is the first case in the literature in which chronic tubulointerstitial nephritis has been reported concomitant with a disseminated sporotrichosis. One of the concerns of this diagnosis is the self-administration of NSAIDs; being at the beginning of the symptoms reported by the patient (3 months before admission) and at a low dose and short period of time, the probability of being drug-induced is low [50].

## Data Availability

Additional information related to this case is available from the corresponding author upon reasonable request.
